# A Double Fluorescent Staining Method Increases the Sensitivity of the Cytokinesis-Block Micronucleus Assay

**DOI:** 10.3390/mps8010003

**Published:** 2025-01-04

**Authors:** Ion Udroiu

**Affiliations:** Dipartimento di Scienze, Università degli Studi “Roma Tre”, 00146 Rome, Italy; ion.udroiu@uniroma3.it

**Keywords:** in vitro, microscopy, mutagenic, specificity

## Abstract

The micronucleus test is one of the most popular genotoxicity assays. In order to avoid underestimation of micronuclei frequencies by counting non-replicating cells, the cytokinesis-blocked micronucleus test has been developed. In this technique, only binucleated cells are scored. One underestimated problem is the potential difficulty in discriminating binucleated from mononucleated cells when using DAPI staining, i.e., the possibility that two neighboring mononucleated cells could be mistaken for a binucleated one. The new protocol presented here comprises the addition of acridine orange in order to stain the cytoplasm (in addition to DAPI to stain nuclei and micronuclei). This new technique can increase the sensitivity of the cytokinesis-blocked micronucleus test and avoid underestimation of micronuclei frequencies, an important issue when high doses are employed.

## 1. Introduction

Micronuclei (MN) are extra-nuclear bodies that form during mitosis and contain a chromosome fragment or a whole chromosome that has not migrated with one of the two daughter nuclei [1]. These inclusions may be found in any kind of cell, both somatic and germinal. Being the product of unrepaired DNA double-strand breaks (acentric fragments) or chromosome malsegregation, they are indicative of genotoxic damage suffered by the cell.

MN have been known in hematology as Howell–Jolly bodies for 120 years [2]. Evans et al. [3], using gamma-irradiated *Vicia faba* roots, performed the first dose–response curves of MN. Later, the study of the variations in MN frequencies began to develop into a standardized assay, leading to the “micronucleus test” [4]. Because of its ease to be performed, it became a very popular assay, both in vivo and in vitro.

Following genotoxic damage, some cells may undergo cell cycle block [1]. Since the formation of an MN requires the cell to undergo mitosis, the MN frequencies are underestimated if non-replicating cells are counted. In fact, non-replicating cells were counted together (and as) replicating cells with no MN, thus “diluting” the population of micronucleated cells and lowering the MN frequency.

In order to avoid this, Fenech and Morley [5] developed the cytokinesis-blocked MN (CBMN) test. In this protocol, cytochalasin-B is used to block cytokinesis (i.e., the division of daughter cells) without interfering with mitosis. In this way, following one nuclear division, the replicating cell forms a binucleated cell. By scoring only binucleated cells, false negatives are excluded, i.e., those cells that suffer genotoxic damage, leading to the formation of an acentric fragment, which, in turn, does not contribute to an MN because the cell is blocked before mitosis.

For many years, the CBMN test has been performed using a nonspecific stain, i.e., Giemsa or May–Grünwald–Giemsa. Later, several authors began to employ DNA-specific stains, such as 4′,6-diamidino-2-phenylindole (DAPI) [6,7,8], also for automated scoring [9]. Staining with DAPI is time-saving (a few minutes vs. one hour for the May–Grünwald–Giemsa protocol) and more specific in many cases. For example, in exfoliated epithelial cells, keratin bodies may be misinterpreted as MN with nonspecific DNA stains [10].

One underestimated problem is the potential difficulty in discriminating binucleated from mononucleated cells when using DAPI staining. Indeed, as only nuclei are visible (and not cytoplasms), two neighboring mononucleated cells can be mistaken for a binucleated one. This is particularly true for adherent cell lines, whereas it is less important (but not absent) for lymphocytes or other cells growing in suspension. Nonetheless, if tetranucleated lymphocytes (produced by two subsequent cell divisions) are present, these could be mistaken for two neighboring binucleated cells. Above all, misinterpretation of BNCs can become a serious issue when high doses of genotoxic agents are used and proliferation is depressed, affecting the detection of MN.

In view of this, it can be said that performing the CBMN test with only a DNA-specific stain (e.g., DAPI) can yield false results and lower the sensitivity of this assay. This is particularly true with high doses of genotoxic agents; indeed, it has been known for many years that, in dose–response studies with X-rays, above some doses, the frequency of MN starts to diminish [1]. Since MN frequencies are scored per BNC, they should be independent of the yield of BNCs (which diminishes as the dose increases). Therefore, this phenomenon is quite a paradox. An explanation has been provided with the hypothesis that severely damaged cells are blocked before mitosis, and, therefore, although harboring acentric fragments, these cannot be converted into MN, thus lowering the frequencies of MN per BNC [1]. Another factor, however, could be the abovementioned misidentification of two neighboring mononucleated cells as a BNC. This can lead to an overestimation of BNCs and, more importantly, an underestimation of MN frequencies. The present paper proposes a simple staining protocol to avoid this issue.

## 2. Materials and Methods

Early passage (3–4) Human Lens Epithelial Cells B3 (ATCC, USA) were grown in Eagle’s minimal essential medium with Earle’s salt (Euroclone, Milan, Italy) supplemented with 20% fetal bovine serum (Euroclone, Milan, Italy), with 10,000 units/mL of penicillin, 10 mg/mL of streptomycin (Corning, Corning, NY, USA), and 1% non-essential amino acid (Euroclone, Milan, Italy). The cells were grown in an incubator at 37 °C, with 95% relative humidity and 5% CO_2_.

Irradiation was performed with radiological equipment (6 mA; 0.3 mm Cu filter; Gilardoni, Lecco, Italy), applying 168 kVp peak potential (producing 74 keV mean photon energy).

Twenty-four hours before irradiation, 50,000 cells were seeded onto slides inside 35 mm Petri dishes. Cytochalasin-B (final concentration: 3 μg/mL in DMSO, Sigma-Aldrich, Burlington, MA, USA) was added immediately after irradiation. After 24 h, slides were fixed with methanol and stained with 1 μg/mL of DAPI (4′,6-diamidino-2-phenylindole, Sigma-Aldrich, USA) and acridine orange (Sigma-Aldrich, USA). A stock solution (1 mg/mL) of acridine orange in deionized water was kept at +4 °C in dark. For slide staining, fresh solutions were obtained by diluting aliquots of the stock solution in Sørensen’s buffer at pH 6.8 to a final concentration of 20 μg/mL.

Micronuclei (MN) were scored at 63× magnification using a Zeiss Axiophot Z2 (Carl Zeiss, Jena, Germany) microscope with FITC (488 nm excitation; 525 nm barrier) and/or DAPI filter (359 nm excitation; 441 nm barrier). MN frequencies were assessed scoring 1000 binucleated cells (BNCs) for each sample. The percentage of binucleated cells was evaluated scoring 1000 cells (mono- and binucleated). For each treatment, at least 3 independent replicates were used.

Each sample was analyzed twice: the first time only with DAPI filter, the second time with FITC and DAPI filters. In the latter case, each visual field was first examined with FITC filter (scoring the number of mono- and binucleated cells). Then, DAPI filter was used to assess the presence of MN in the binucleated cells.

*T* test was used to compare MN frequencies and BNC percentages of the different samples. For each dose, comparison was conducted between results obtained with DAPI alone and those with DAPI and acridine orange. The level of significance was established at *p*  <  0.05.

## 3. Results

**Figure 1:**
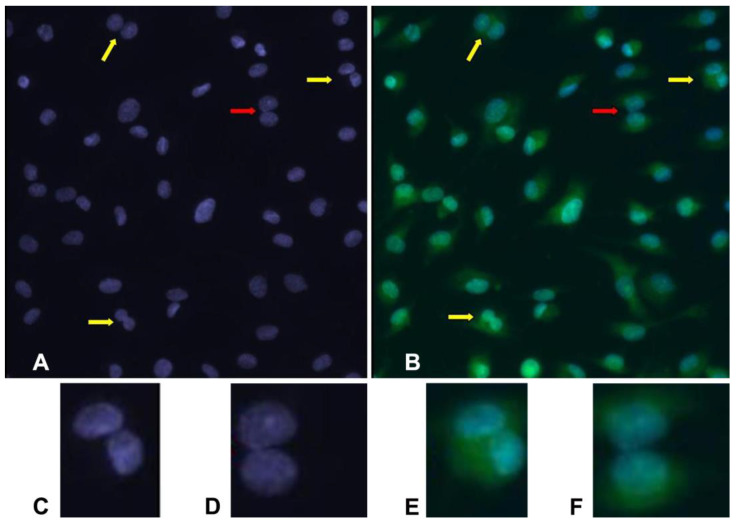
Representative images of binucleated cells: (**A**) cells analyzed with DAPI alone; (**B**) cells analyzed with DAPI and acridine orange. Yellow arrows show true binucleated cells. Red arrow shows two neighboring nuclei that can be interpreted as a binucleated cell if analyzed with DAPI alone (**A**), but they are revealed as two mononucleated cells by acridine orange staining (**B**). (**C**,**D**) Cells analyzed with DAPI alone at higher magnification. (**E**,**F**) Same cells analyzed with DAPI and acridine orange, revealing that (**C**,**E**) is a binucleated cell and (**D**,**F**) are two neighboring mononucleated cells. Images (**A**,**B**) are at 63× magnification; images (**C**–**F**) are at 20×.

In samples analyzed with both DAPI and acridine orange, BNCs are easily recognized. This contrasts with DAPI alone, in which some situations are dubious. Comparing some images, it is evident that cells misinterpreted as a binucleated one are revealed to be two neighboring mononucleated cells by acridine orange staining (Figure 1).

**Figure 2:**
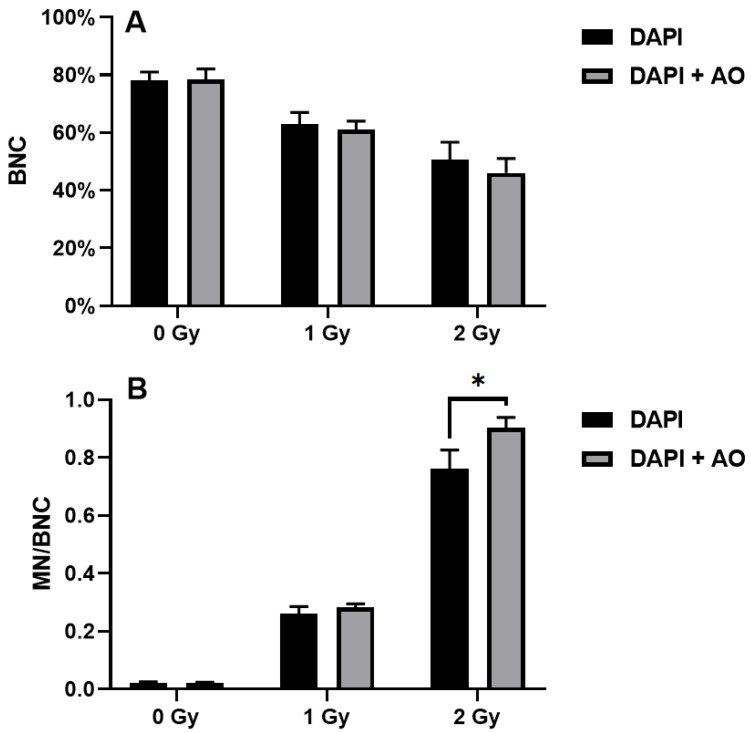
Influence of staining on the micronucleus test results: (**A**) percentages of binucleated cells; (**B**) frequencies of micronucleated cells. BNCs: binucleated cells. MN: micronuclei. AO: acridine orange. Gy: gray. Asterisk represents significant difference (*p* < 0.05).

Probably due to the presence of false positives, the percentages of BNCs demonstrated to be higher when scoring was performed using DAPI alone compared to DAPI and acridine orange (Figure 2A). In the unirradiated samples, the percentages of BNCs were 78 ± 3% and 78.3 ± 3.8% when the samples were scored with DAPI alone and DAPI and acridine orange, respectively. In the samples irradiated with the 1 Gy X-ray, the percentages of BNCs were 63 ± 4% and 61 ± 3% when the samples were scored with DAPI alone and DAPI and acridine orange, respectively. Finally, the 2 Gy-irradiated samples showed 50.7 ± 6% and 46 ± 5% BNCs when the samples were scored with DAPI alone and DAPI and acridine orange, respectively. Indeed, the difference between the two techniques increased with increasing dose of X-ray.

Also, the differences in micronucleated BNCs increased with increasing dose of X-ray (Figure 2B). In this case, the MN frequencies were lower when scoring was performed using DAPI alone compared to DAPI and acridine orange. In the unirradiated samples, the frequencies of micronucleated BNCs were 0.021 ± 0.004 and 0.021 ± 3.0.003 when the samples were scored with DAPI alone and DAPI and acridine orange, respectively. In the samples irradiated with the 1 Gy X-ray, the frequencies of micronucleated BNCs were 0.261 ± 0.025 and 0.281 ± 0.014 when the samples were scored with DAPI alone and DAPI and acridine orange, respectively. Finally, the 2 Gy-irradiated samples showed 0.761 ± 0.065 and 0.902 ± 0.037 MN per BNC when the samples were scored with DAPI alone and DAPI and acridine orange, respectively. The difference was statistically significant in those samples irradiated with 2 Gy (*p* = 0.03).

## 4. Discussion

The micronucleus test is perhaps the most widely used genotoxic assay, both in vivo and in vitro [11,12]. Being one of the most sensitive tests to detect genotoxicity [13], it has been widely used to predict the potential carcinogenicity of chemical compounds [14], as well as radiation triage and biological dosimetry [15]. However, genotoxic agents can (and often do) exert indirect effects that go beyond sole damage to DNA. In fact, DNA damage induces a cellular response that comprises not only activation of the DNA repair machinery but also activation of checkpoints that delay cell cycle progression or even block it altogether (i.e., determining senescence) [16]. With the cell cycle delayed or even blocked, the number of cells having undergone mitosis during the experiment decreases, with the magnitude of the decrease depending not only on the dose used (and, therefore, on the extent of DNA damage) but also on the cell type [1].

Inhibition of mitosis by genotoxic agents, however, can lead to underestimation of MN frequencies since non-replicating cells (which cannot contribute to MN even if containing acentric fragments) are scored together with replicating ones. In order to avoid this problem, the CBMN has been developed [5]. When using DNA-specific fluorescent staining, the cytoplasm is not visible, leading to the possibility that two neighboring mononucleated cells can be mistaken for a binucleated one. In this way, when mononucleated cells (which cannot contain MN because they have not passed through mitosis) are scored as a BNC, the frequency of MN is lowered. These “false binucleated cells” become a serious issue when high doses (for example of radiation), leading to a low ratio of binucleated to mononucleated cells [1], are used. In the case of automated scoring, the number of false binucleated cells is so high that a trained scorer must review the acquired images and reclassify the incorrectly classified cells, seriously slowing down the automated protocol [17]. Summing up, using only a DNA-specific stain for the CBMN test can lead to an overestimation of BNCs and, more importantly, an underestimation of MN frequencies. This is particularly true when high doses of genotoxic agents (both chemicals and ionizing radiation) are used, i.e., when proliferation is depressed and BNCs become less abundant.

The new protocol presented here comprises the addition of a single simple new step, the addition of acridine orange, in order to stain the cytoplasm. Acridine orange is a fluorescent dye that emits at different wavelengths depending on if it binds single-stranded RNA or double-stranded DNA [18]. It could be objected that, as acridine orange reacts both with RNA and DNA (thus staining both cytoplasm and nuclei/micronuclei, respectively), adding DAPI is useless. In effect, some authors performed the CBMN test with acridine orange alone [19,20]. Nonetheless, one disadvantage of acridine orange is that this fluorochrome is subject to fading [19]. In particular, after a few minutes of exposure to the microscope light, the staining remains fluorescent, but differences between RNA and DNA disappear. In this way, the cell becomes almost uniformly stained and the MN become almost undistinguishable. For this reason, the addition of DAPI (which is also a very simple step in the protocol) is preferable. Evidently, the use of acridine orange in the present protocol is intended just as a means to stain the whole cell no matter its differential staining of RNA or DNA. Indeed, any other stain for RNA could be used, but, as far as I know, acridine orange is the cheapest and easiest to find.

Finally, it should be added that, in the present study, only adherent cells were used. The differences between the results obtained with DAPI alone and DAPI and acridine orange could vary when using cells growing in suspension (such as lymphocytes). However, the risk of misinterpreting two neighboring mononucleated cells as a BNC is much higher when using adherent cells, and, thus, adopting the new protocol seems to be more important for such cells.

## 5. Conclusions

In conclusion, this new protocol, comprising the easy-to-complete staining with DAPI and acridine orange, can increase the sensitivity of the CBMN test and avoid the underestimation of MN frequencies, an important issue when high doses are employed.

## Data Availability

No additional data were created or analyzed in this study. Data sharing is not applicable to this article.
